# Laurinterol from *Laurencia johnstonii* eliminates *Naegleria fowleri* triggering PCD by inhibition of ATPases

**DOI:** 10.1038/s41598-020-74729-y

**Published:** 2020-10-20

**Authors:** Iñigo Arberas-Jiménez, Sara García-Davis, Aitor Rizo-Liendo, Ines Sifaoui, María Reyes-Batlle, Olfa Chiboub, Rubén L. Rodríguez-Expósito, Ana R. Díaz-Marrero, José E. Piñero, José J. Fernández, Jacob Lorenzo-Morales

**Affiliations:** 1 Instituto Universitario de Enfermedades Tropicales y Salud Pública de Canarias (IUETSPC), Universidad de La Laguna (ULL), Avda. Astrofísico F. Sánchez, s/n, 38203 La Laguna, Tenerife Spain; 2 Departamento de Obstetricia y Ginecología, Pediatría, Medicina Preventiva y Salud Pública, Toxicología, Medicina Legal Y Forense Y Parasitología, Universidad de La Laguna (ULL), Avda. Astrofísico F. Sánchez, s/n, 38206 La Laguna, Tenerife Spain; 3 Red de Investigación Colaborativa en Enfermedades Tropicales (RICET), Madrid, Spain; 4 Instituto Universitario de Bio-Orgánica Antonio González (IUBO AG), Universidad de La Laguna (ULL), Avda. Astrofísico F. Sánchez, 2, 38206 La Laguna, Tenerife Spain; 5 Laboratoire Matériaux-Molécules Et Applications, La Marsa, University of Carthage, Tunis, Tunisia; 6 Departamento de Química Orgánica, Universidad de La Laguna (ULL), Avda. Astrofísico F. Sánchez, s/n, 38206 La Laguna, Tenerife Spain

**Keywords:** Parasitology, Pathogens

## Abstract

Primary amoebic encephalitis (PAM) is a lethal disease caused by the opportunistic pathogen, *Naegleria fowleri*. This amoebic species is able to live freely in warm aquatic habitats and to infect children and young adults when they perform risk activities in these water bodies such as swimming or splashing. Besides the need to increase awareness of PAM which will allow an early diagnosis, the development of fully effective therapeutic agents is needed. Current treatment options are amphotericin B and miltefosine which are not fully effective and also present toxicity issues. In this study, the in vitro activity of various sesquiterpenes isolated from the red alga *Laurencia johnstonii* were tested against the trophozoite stage of a strain of *Naegleria fowleri*. Moreover, the induced effects (apoptotic cell death) of the most active compound, laurinterol (**1**), was evaluated by measuring DNA condensation, damages at the mitochondrial level, cell membrane disruption and production of reactive oxygen species (ROS). The obtained results demonstrated that laurinterol was able to eliminate the amoebae at concentrations of 13.42 ± 2.57 µM and also to induced programmed cell death (PCD) in the treated amoebae. Moreover, since ATP levels were highly affected and laurinterol has been previously reported as an inhibitor of the Na^+^/K^+^-ATPase sodium–potassium ion pump, comparison with known inhibitors of ATPases were carried out. Our results points out that laurinterol was able to inhibit ENA ATPase pump at concentrations 100 times lower than furosemide.

## Introduction

*Naegleria fowleri* species, also known as brain eating amoebae is an opportunistic pathogenic amoebae which is able to colonize the central nervous system (CNS) causing an infection known as primary amoebic meningoencephalitis (PAM)^1–3^. The disease affects mainly healthy children and young adults who have reported previous nasal exposure to amoebae-contaminated water during the practice of risk activities such as diving, splashing, among others, in warm water bodies. Moreover, the incidence of PAM since the year 2000 has seen an increase worldwide as it has been recently reported^4^.

Primary amoebic encephalitis was first reported in 1965 in Australia^5^. Since then, PAM cases have been recorded worldwide and it is usually described as a rare disease with only 431 cases available in the literature^4^.

*Naegleria fowleri* infects humans by passing the nerves and entering the cribiform plate. After that, the amoebae have free entrance to the brain causing inflammation and necrosis^2,6–8^. Hence, the fulminant nature of this infection which normaly manifests in the patient with symptons such as intense headache, temperature, seizures and stiff neck. In the final stage of the disease, patients have been reported to suffer from hallucinations and paralysis previous reaching coma and death^6–8^. Furthermore, the average time of symptoms appearance after infection starts 1–9 days after exposure to contaminated water sources whereas patient death average is 1–18 after symptoms begin^4,9^.

Regarding diagnosis of PAM, it is often undertaken *post-mortem* because the clinical symptons are not specific. Therefore, late diagnostics correlates to delayed treatment which commonly ends with the lost of the patients^1,10,11^. Current therapeutic options involves a combination of drugs which includes amphotericin B, azithromycin, rifampin, azoles and lately miltefosine with or without the combination of hypothermia^6,12,13^. Even though, recent cases of PAM treatment have been reported to be successful^14–16^, there is still an urgent need to find novel anti-amoebic agents which are able to eliminate the pathogen also causing low toxicity.

Natural products have been used for the treatment of different parasitic diseases, among them artemisinin, quinine and ivermectin are examples of important antiparasitic compounds from this origin^17–19^. Moreover, and specially in the case of antiamoebic compounds, algae have recently revealed their potential as a source of compounds with therapeutic potential^20,21^.

The genus *Laurencia* is one of the richest sources of active compounds among red algae which have also previously revealed metabolites presenting antiprotozoal and antiparasitic properties^22–24^. However, no activity of these metabolites has been previously reported against *Naegleria fowleri*.

In the present study, previously identified sesquiterpenes from *Laurencia johnstonii* species in our group were re-isolated and evaluated against the trophozoite stage of a type strain of *Naegleria fowleri*. Furthermore, the most active molecule was further investigated in order to establish the mechanisms of programmed cell death induced in the treated amoebae.

## Results

**Figure 1:**
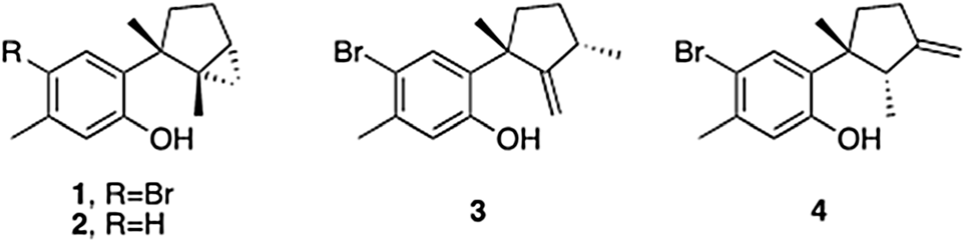
Structure of phenolic sesquiterpenes isolated from *Laurencia* species.

A series of natural phenolic sesquiterpenes (**1**–**3**) (Fig. 1) obtained from specimens of *Laurencia jonhstonii* were selected to screen their in vitro activities against *Naegleria fowleri*. The obtained results which are shown in Table 1, revealed that laurinterol (**1**) was the most active molecule with an IC_50_ of 13.42 ± 2.57 µM. Therefore this compound was selected for further studies focused on the study of the induced mechanisms of programmed cell death in the treated amoebae.

**Table 1:** Effect of phenolic sesquiterpenes **1**–**3**, isolated from *Laurencia johnstonii*, against *Naegleria fowleri* ATCC 30,808 (IC_50_) and murine macrophages J774.A1 cell line (CC_50_).

Sample	IC_50_ (µM)	CC_50_ (µM)
Laurinterol (**1**)	13.42 ± 2.57	80.11 ± 7.79
Debromolaurinterol (**2**)	18.76 ± 4.03	70.13 ± 12.94
Isolauritenol (**3**)	28.18 ± 0.13	24.74 ± 2.39
Amphotericin B	0.12 ± 0.03	> 200

### Laurinterol eliminates *Naegleria fowleri* trophozoites at low concentrations and presents low cytotoxicity levels

**Figure 2:**
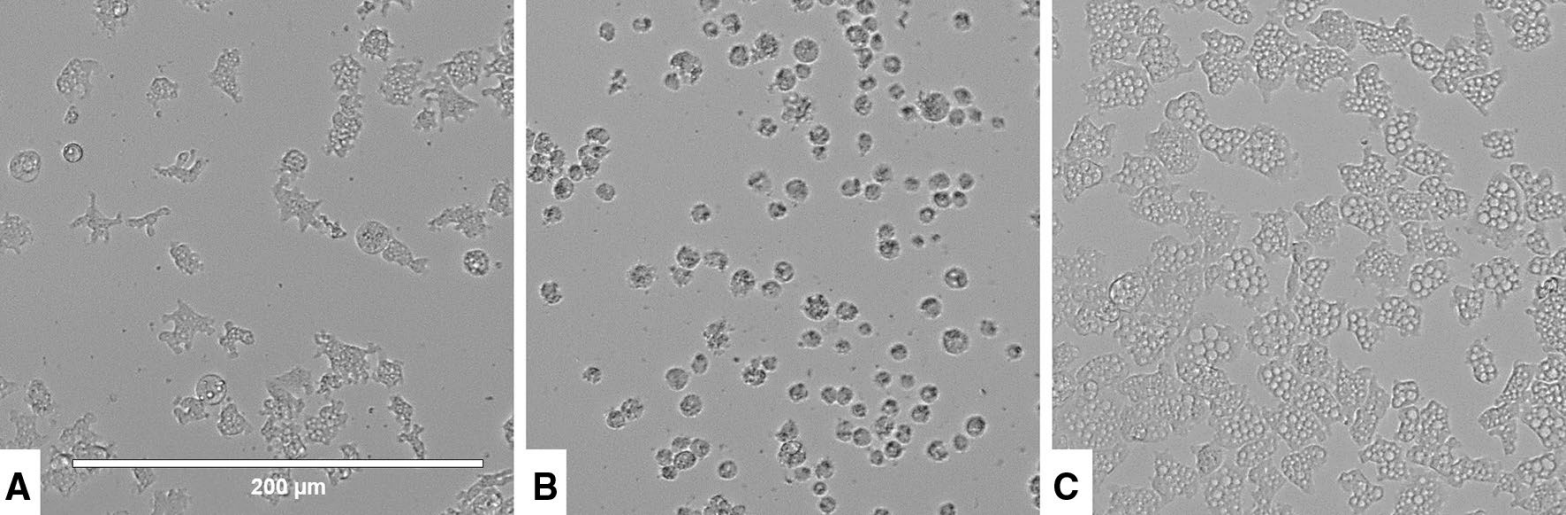
*Naegleria fowleri* trophozoite incubated with the IC_50_ (**A**) and IC_90_ (**B**) of laurinterol (**1**) (48 h). Images are representative of the cell population observed in the performed experiments. Negative control (**C**). Images (20 ×) were obtained using an EVOS FL Cell Imaging System AMF4300, Life Technologies, Madrid, Spain.

Laurinterol eliminated *N. fowleri* trophozoites when incubated in vitro in a dose dependent way. The obtained results using the Alamar Blue colorimetric assay (described in the “Methods” section) allowed us to establish an IC_50_ value of 13.42 ± 2.57 µM(Fig. 2). Furthermore, toxicity assays against the murine macrophage J774.A1 cell line yielded a CC_50_ of 80.11 ± 7.79 µM. Therefore, the toxicity was considered low when compared to the obtained *Naegleria* inhibitory concentrations of laurinterol.

**Figure 3:**
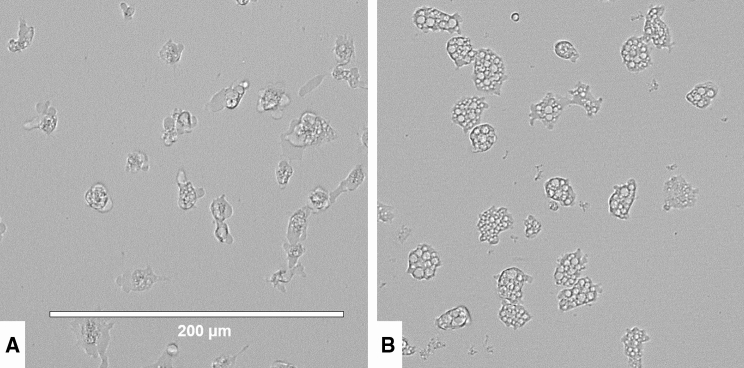
Effect of the IC_90_ of laurinterol (**1**) in *Naegleria fowleri* trophozoite after 1 h of incubation (**A**). Images are representative of the cell population observed in the performed experiments. Negative control (**B**). Images (20 ×) were obtained using an EVOS FL Cell Imaging System AMF4300, Life Technologies, Madrid, Spain.

Moreover, amoebic forms were observed to present morphological changes such as loss of complexity in the cytoplasm and lack of cellular structure as well as cell volume (Fig. 3), at 1 h time of incubation in the presence of laurinterol at the IC_90_ (101.69 ± 0.67 µM).

### Laurinterol-treated amoebae stained positive in the double stain assay

**Figure 4:**
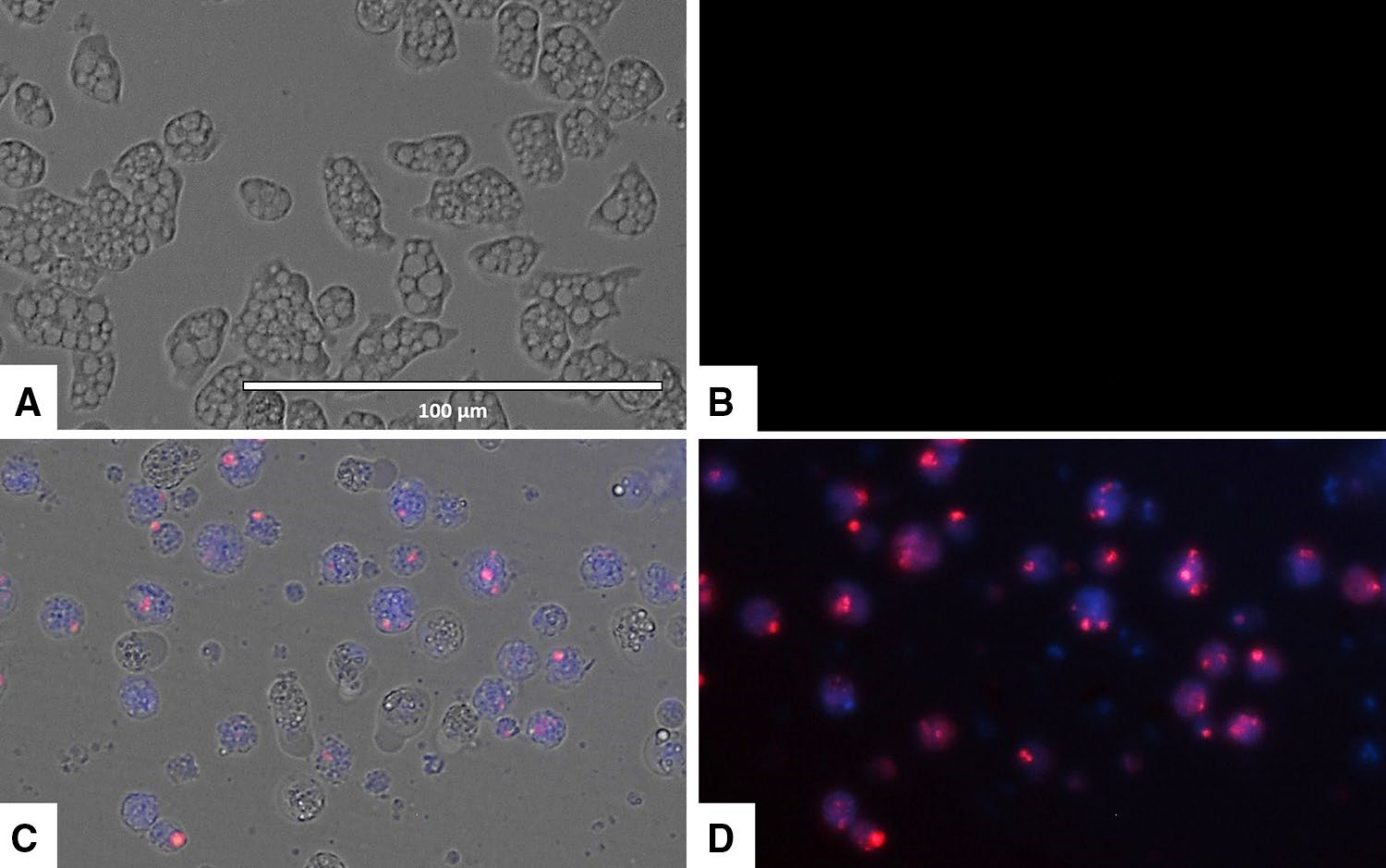
*Naegleria fowleri* trophozoites incubated with IC_90_ of laurinterol (**1**) for 24 h. Hoechst stain is different in control cells, where uniformly faint-blue nuclei are observed (**A**,**B**), and in treated cells, where the nuclei are bright blue (**C**,**D**). Red fluorescence corresponds to the propidium iodide stain. Images (40 ×) are showing chromatin condensation (blue) in treated cells. Images are representative of the cell population observed in the performed experiments. Images were obtained using an EVOS FL Cell Imaging System AMF4300, Life Technologies, Madrid, Spain.

**Figure 5:**
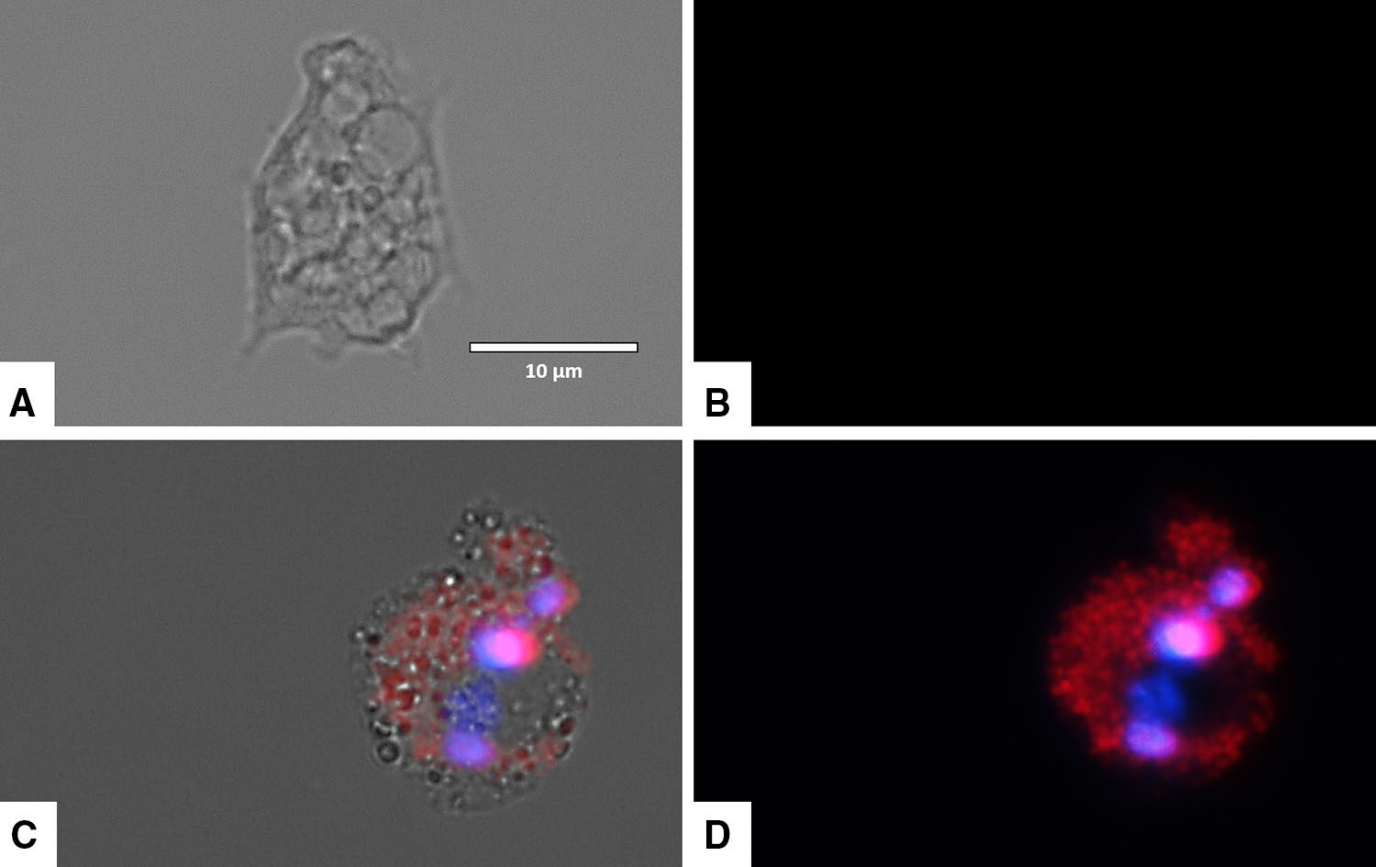
Higher magnification of Fig. 4. Images (100 ×) are showing late apoptotic cell with high blue and red fluorescence. Images are representative of the cell population observed in the performed experiments. Images were obtained using an EVOS FL Cell Imaging System AMF4300, Life Technologies, Madrid, Spain.

Laurinterol induced chromatic condensation of treated amoebae at a concentration of IC_90_. Treated amoebae showed the typical bright-blue stained nuclei at 24 h post incubation with the molecule as shown in Figs. 4 and 5.

### Laurinterol-treated amoebae showed plasma membrane permeability

**Figure 6:**
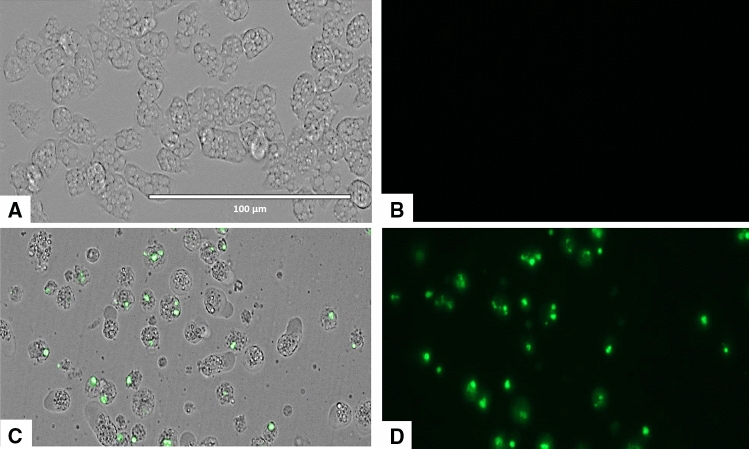
Permeation of the *Naegleria fowleri* plasma membrane to the vital dye SYTOX green caused by addition of laurinterol (**1**) at IC_90_ after 24 h (**C**,**D**). Negative Control (**A**,**B**). Images (40 ×) are representative of the cell population observed in the performed experiments. Images were obtained using an EVOS FL Cell Imaging System AMF4300, Life Technologies, Madrid, Spain.

**Figure 7:**
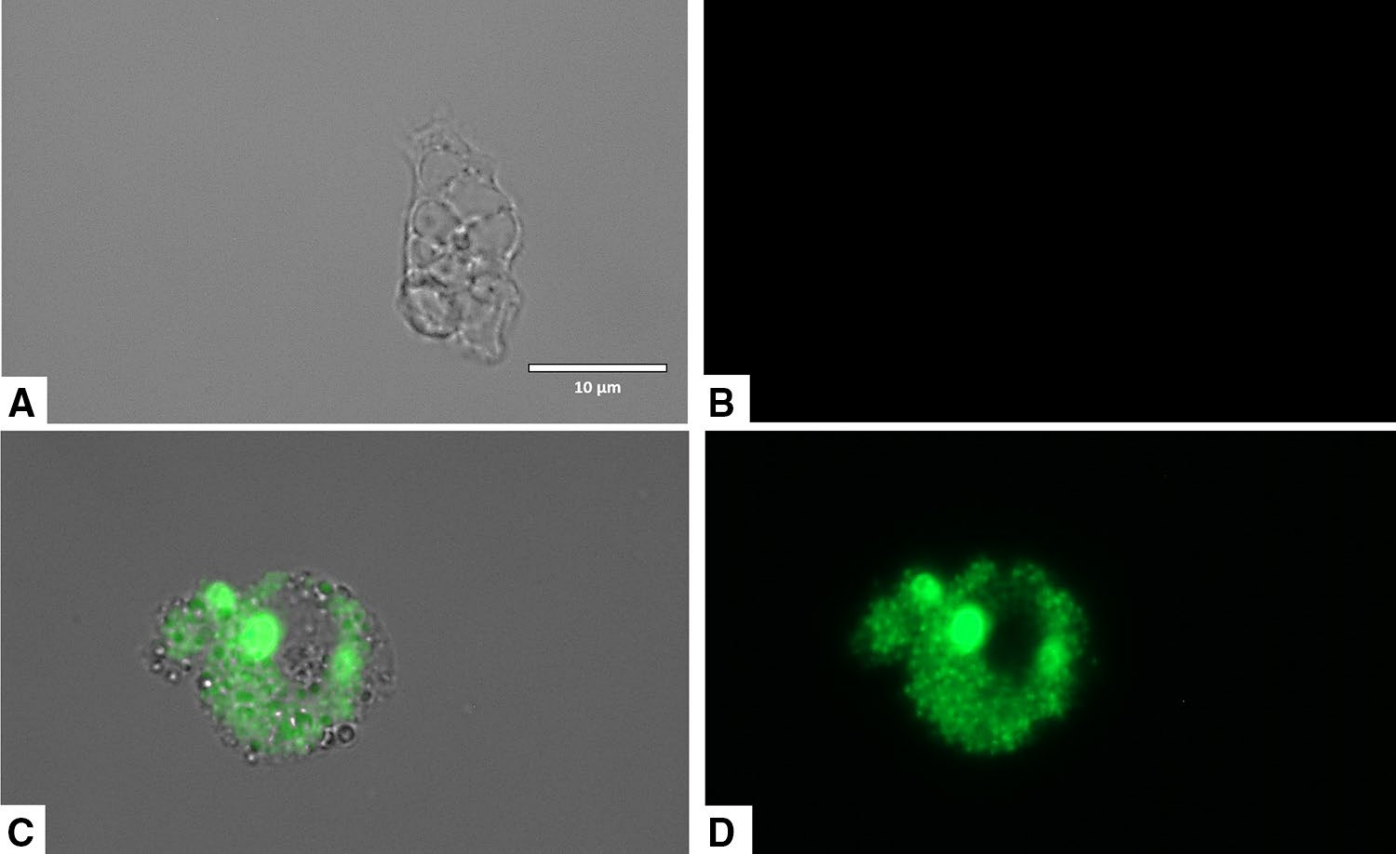
Higher magnification of Fig. 6 (100 ×). Negative control (**A**,**B**). Cells treated with the IC_90_ of laurinterol (**1**) for 24 h. Images were obtained using an EVOS FL Cell Imaging System AMF4300, Life Technologies, Madrid, Spain.

As shown in Figs. 6 and 7, amoebae treated with the IC_90_ of laurinterol experienced plasmatic membrane damage after 24 h of incubation.

### Laurinterol causes mitochondrial malfunction in treated amoebae

**Figure 8:**
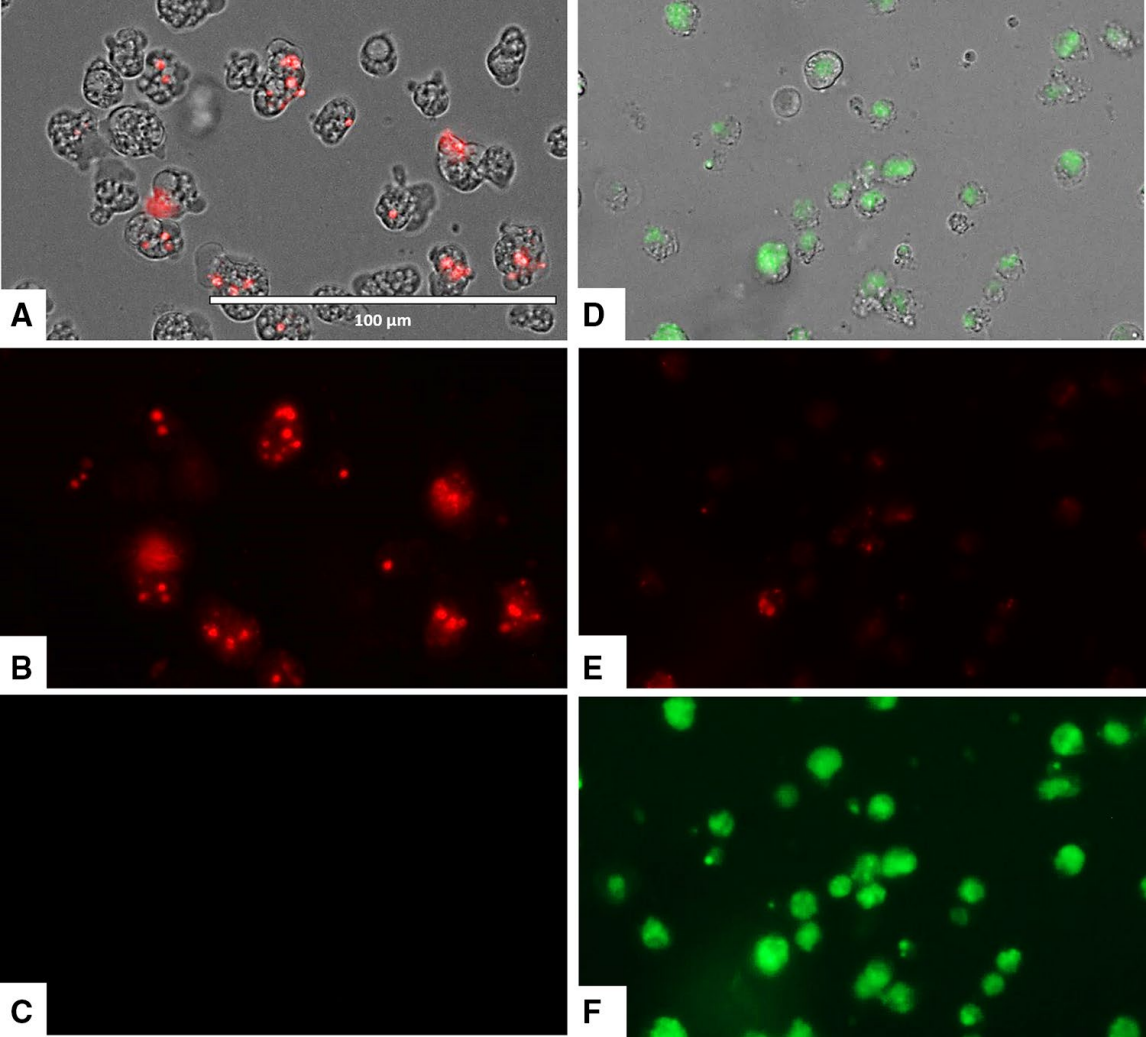
The effect of laurinterol (**1**) on the mitochondrial potential, JC-1 dye accumulates in the mitochondria of healthy cells as aggregates and emit a red fluorescence (**A**–**C**: Negative control). Cells treated with the IC_90_ of laurinterol (**1**) for 24 h, emit green fluorescence (**D**–**F**): due to the decrease in mitochondrial membrane potential, the JC-1 dye remained in the cytoplasm in its monomeric form and emit in this case a green fluorescence. (Images are representative of the population of treated cells 40 ×). Images were obtained using an EVOS FL Cell Imaging System AMF4300, Life Technologies, Madrid, Spain.

**Figure 9:**
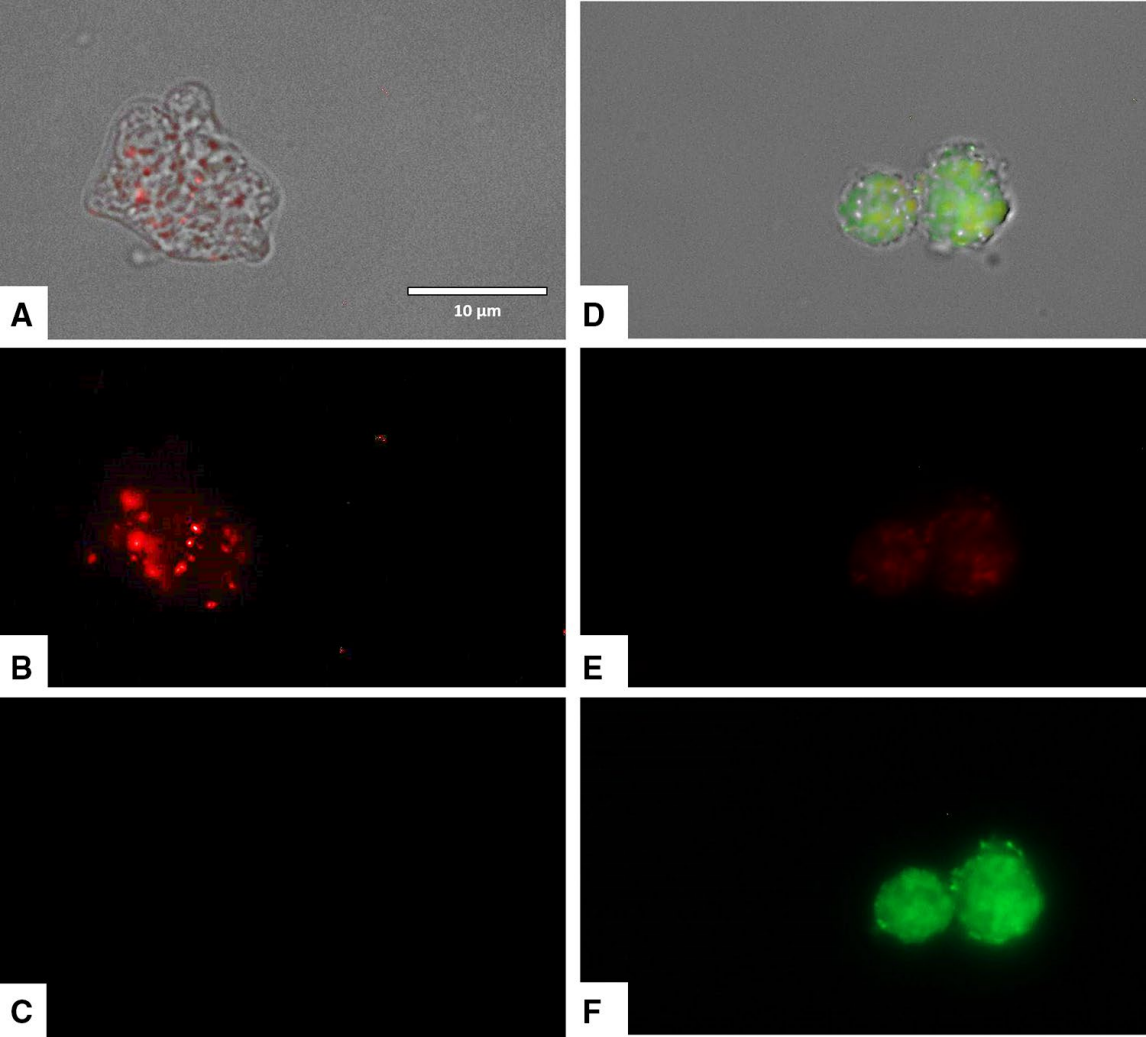
Higher magnification of Fig. 8 (100 ×). Images were obtained using an EVOS FL Cell Imaging System AMF4300, Life Technologies, Madrid, Spain.

Laurinterol induced changes on the mitochondrial potential of *Naegleria fowleri* treated trophozoites since the JC-1 dye remained in the cytoplasm in its monomeric form, shown as green fluorescence (Figs. 8, 9). Furthermore, the mitochondrial damage was also checked by measuring the generated ATP levels in 24 h. The observed results showed that laurinterol IC_90_ treated amoebae presented a 97.36% decrease of ATP levels when compared to untreated cells. Moreover, since laurinterol has been reported as an inhibitor of the Na^+^/K^+^-ATPase sodium–potassium ion pump^25^, activity assays of known inhibitors including ouabain and furosemide were carried out. Amoebae showed to be ouabain-insensitive even at high concentrations of this product whereas furosemide showed an IC_50_ of 0.71 ± 0.04 mM.

### Laurinterol increases reactive oxygen species (ROS) levels in *Naegleria fowleri*

**Figure 10:**
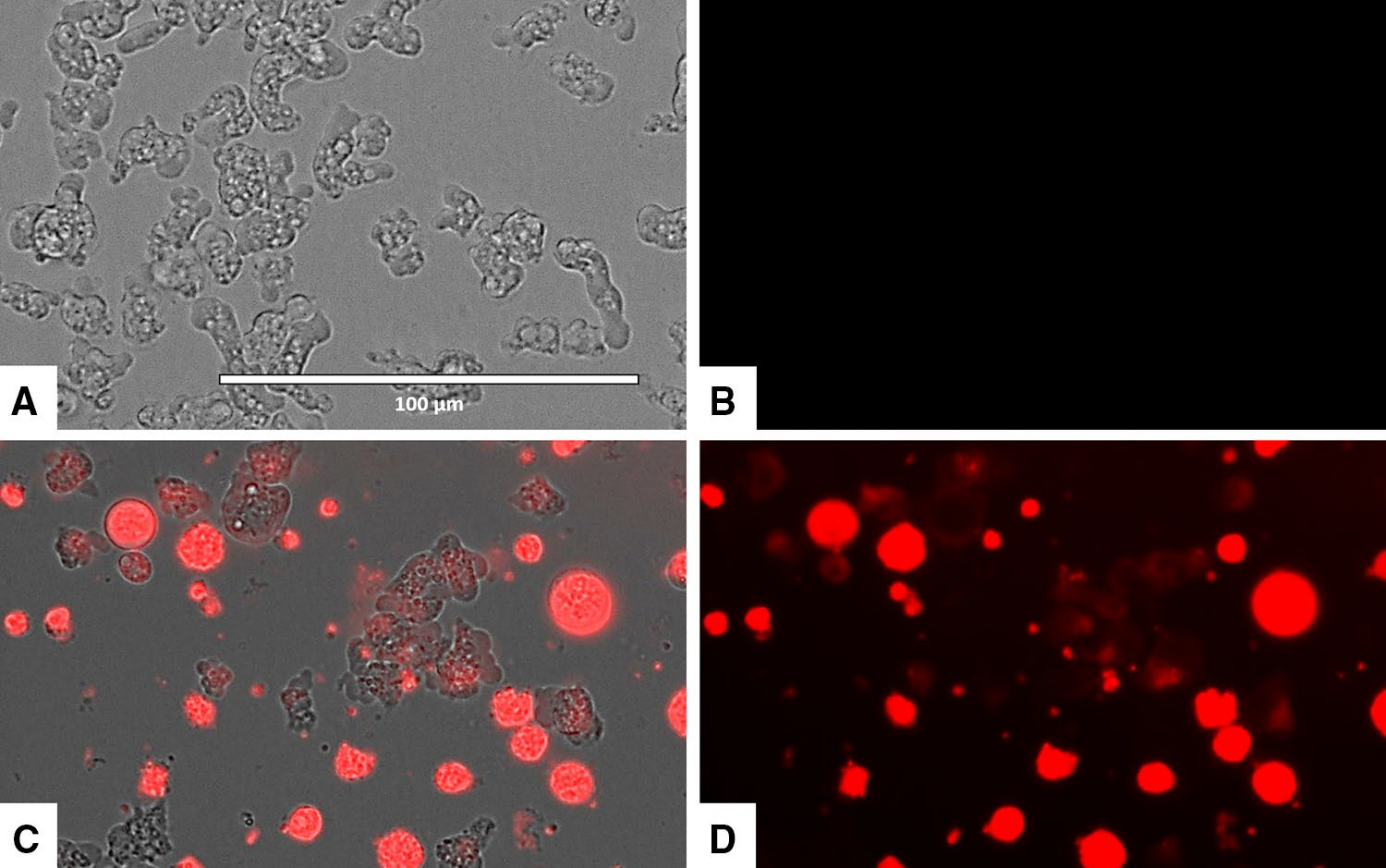
Increase levels of ROS in *Naegleria fowleri* caused by addition of laurinterol (**1**) at IC_90_ after 24 h (**C**,**D**). Laurinterol (**1**) was added to cells (10^5^ cells/mL) and exposed to CellROX Deep Red (5 μM, 30 min) at 37 °C in the dark. Negative Control (**A**,**B**): cells exposed to CellROX Deep Red in absence of laurinterol (**1**). Images (40 ×) are representative of the cell population observed in the performed experiments. Images were obtained using an EVOS FL Cell Imaging System AMF4300, Life Technologies, Madrid, Spain.

**Figure 11:**
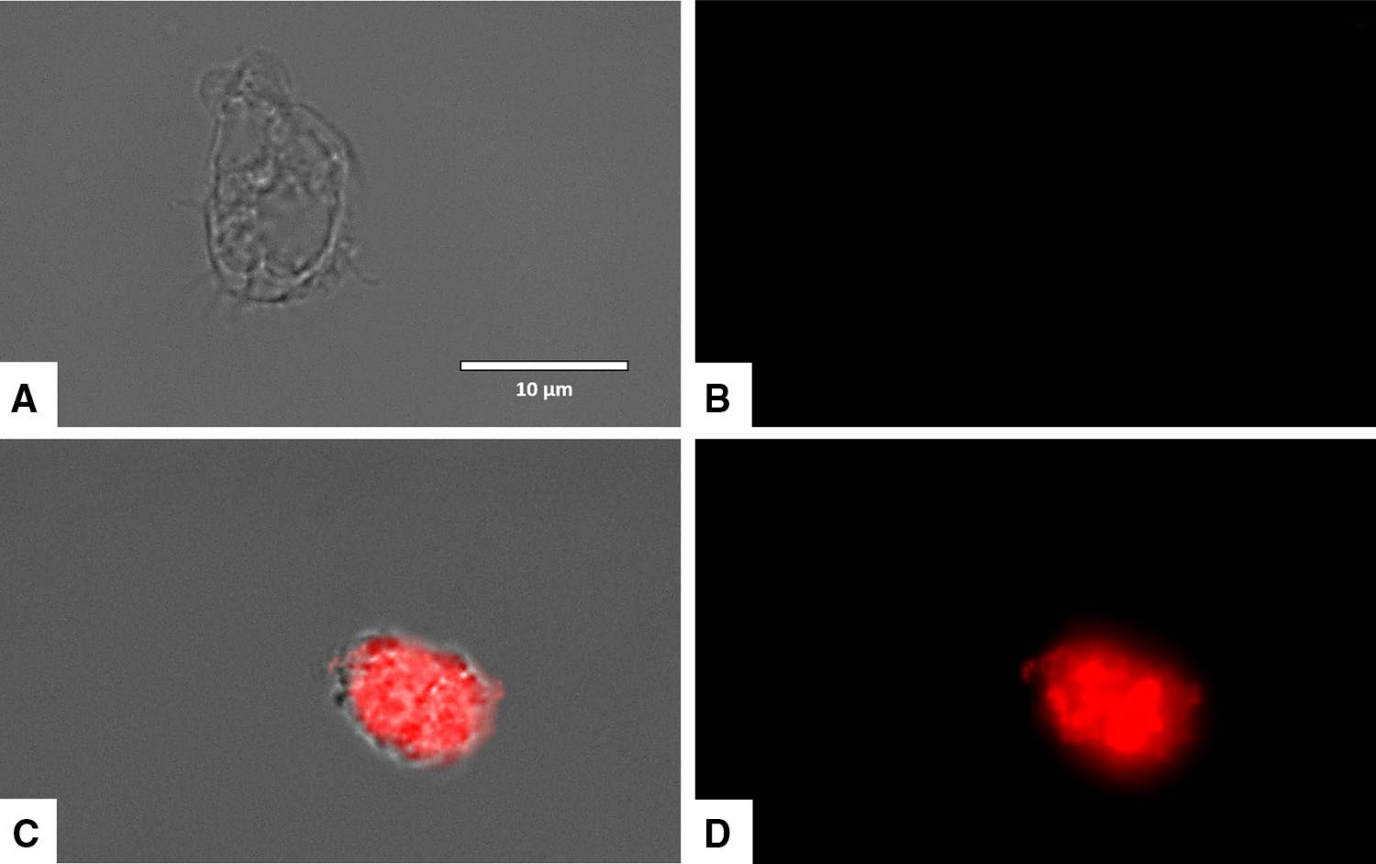
Higher magnification of Fig. 9 (100 ×). Negative control (**A**,**B**). cells treated with the IC_90_ of laurinterol (**1**) for 24 h. Images were obtained using an EVOS FL Cell Imaging System AMF4300, Life Technologies, Madrid, Spain.

*Naegleria fowleri* trophozites treated with laurinterol experienced increased levels of ROS when treated with IC_90_ after 24 h of incubation (Figs. 10 and 11).

## Discussion

The marine environment is an interesting source of bioactive compounds which could be potential novel therapeutic agents against cancer and parasites, among others, as it has been previously described^25^. Furthermore, algae are currently the major source of these type of molecules being *Laurencia* genus one of the richest organisms among the reported ones so far^21,25^. In a recent study by García-Davis^25^, the study of species adapted to live under particular environmental conditions, such as *L. johnstonii*, could be a key factor in the search of potential therapeutic agents from these sources. Interestingly, the species of *L. johnstonii*, endemic from the Pacific coast of the Baja California area, used in this study was reported to present higher contents of laurinterol (**1**) than its counterparts in the Pacific coast^20,25^.

Primary amoebic encephalitis (PAM) caused by *Naegleria fowleri* species, a Free-Living Amoebae which is able to act as an opportunistic pathogen, is a fulminant CNS disease which needs novel, more effective and faster therapeutic options. Furthermore, the current options are not fully effective and lack of availability worldwide creates the need to find alternative options and sources^4,13^.

In this study, we aimed to investigate the potential of some natural sesquiterpenes isolated from *L. johnstonii* against *Naegleria fowleri *in vitro. Regarding the obtained activities against the trophozoite stage, it is important to highlight that the most active tested compound, laurinterol was active at low concentrations although not as active as the commonly used therapeutic agent against *Naegleria* which is amphotericin B (IC_50_ = 0.12 ± 0.03 and CC_50_ ≥ 200 μM)^9^. Nevertheless, laurinterol is a small molecule which could be even synthetized in the laboratory as well as its derivatives^26^. Hence, revisiting the potential use of known small molecules such as this one, at least as anti-amoebic agents, seems to be a good option in this field for drug discovery purposes.

Programmed Cell Death (PCD) and apoptosis-like processes have been previously described in protozoa and in multicellular organisms^27^. The phenomenom of PCD involves morphological and cellular events being the most commonly described ones: chromatin condensation, nuclear DNA fragmentation, cell shrinkage, blebbing, loss of mitochondrial membrane potential, the formation of apoptotic bodies, and the exposure of phosphatidylserine among others^28,29^.

In *Naegleria* genus, PCD events in treated cells with amphotericin B were described by Cardenas-Zuñiga and colleagues for the first time^30^. In this study, the effect of a non-lytic dose of amphotericin B (10 µg/ml) was evaluated in cultures of *N. fowleri* and *N. gruberi*. Among the observed events indicative of PCD, authors reported the presence of blebs, DNA condensation, ROS presence and electron-dense granules^30^.

For the development of novel therapeutic agents, our laboratory has focused on the evaluation of compounds of diverse origin but which are also inducers of PCD/apoptosis-like events, avoiding those molecules that induce necrosis and, hence, inflammation which is a non desirable side effect in the search of antiamoebic agents. In the present study, laurinterol was shown to induce chromatin condensation, plasma membrane damage, mitochondrial disfunction as well as increased levels of ROS. Since these effects were observed even after 24 h time of incubation with laurinterol, we postulate that this molecule could induce apoptosis in *Naegleria* through the intrinsic pathway since the mitochondrial potential was collapsed even at 24 h, as well as ATP level (Figs. 7, 8). PCD is also indicated by the generation of ROS species and condensation of DNA. Moreover, when the macrophage cell line was incubated to laurinterol IC_90_ for 24 h, no signs of PCD events were observed (data not shown). Hence, the observed PCD inductions effects are exclusively induced by this compound in *N. fowleri*.

In addition, the exact mechanism of action of laurinterol in *Naegleria fowleri* still needs further studies to be fully elucidated. Nevertheless, laurinterol has been reported as an inhibitor of Acetylcholinesterases (AChE)^31^. These enzymes play a key role in the human body since they catalyze the breakdown of acetylcholine and of some other choline esters that function as neurotransmitters^32^. In protists, AchE have been postulated to act as biomediators and their action is used by protozoa in processes such as cell to cell communications, reproduction, adhesion or motility. The recently reported genome project data for *Naegleria fowleri* and *Naegleria gruberi* species^33,34^ have made available sequences in the databases (i.e. https://www.uniprot.org/uniprot/D2V1M1) which are compatible to AchE-like enzymes. Therefore, the inactivation of these enzymes could trigger a cascade of events which could be the key to understand the observed morphological changes and PCD related events.

Previous reports indicate that phenolic sequiterpenes laurinterol and debromolaurinterol (**2**) showed inhibitory activity of ouabanin-sensitive dog kidney P Type Na^+^,K^+^-ATPase at IC_50_ of 40 and 400 µM^35,36^. In addition, laurinterol exhibited induction of apoptosis by DNA fragmentation and activation of several caspases in melanoma cells. It was also demonstrated the transcriptional activation of the tumor suppressor gene p53 and the activation of p21 promoter. These studies indicate that laurinterol can induce apoptosis in melanoma cells through a p53-dependent pathway^37^.

The metabolic and histopathological effects of laurinterol on Vero and MCF-7 cell lines to breast cancer explants have been evaluated. A dose-dependent inhibition of the metabolic activity was observed, as well as morphologic and nuclear changes indicative of an apoptotic process. Cultures of human breast cancer explants treated with laurinterol showed a heterogeneous response, probably related with the individual response of each tumor sample. The study supports the cytotoxic and antitumoral effects of **1** both in in vitro cell cultures as in ex vivo organotypic cultures^25^.

On the other hand, allolaurinterol (**4**) (Fig. 1), another phenolic sequiterpene closely related to laurinterol , exhibited inhibition of eIF4A ATPase at low μM concentration. The enzymological study revealed that **4** is an ATP-competitive molecule (K_i_ 3.86 µM), whereas cellular evaluations showed reasonable cytotoxicity against lung cancer A549 and breast cancer MDA-MA-468 cell lines. The compound also showed potent inhibition of helicase activity consistent with its ATPase inhibitory activity with complete inhibition at 100 µM. Thus, this data proved that allolaurienterol is an ATP-competitive molecule most likely binding to the ATP-binding pocket at the interface between the N-terminal and C-terminal domains^38,39^.

In cells, Na^+^,K^+^-ATPase and H^+^-ATPase, two P-type ATPases, are essential to control homeostatic processes. A third P-type ATPase, the K + or Na + efflux ATPase (ENA ATPase), was discovered and, for many years, it was considered to be exclusively a fungal enzyme. ENA ATPase is now known to be also present in bryophytes and protozoa^40–43^. In protozoa, the presence of a ouabain-insensitive Na^+^-ATPase activity was found in *Trypanosoma cruzi* epimastigotes, stimulated by Na^+^, and inhibited by furosemide^41^. The fact that the ENA ATPase is found in almost all fungi, as well as in bryophytes and protozoa has been suggested to be an adaptive mechanism required in these organisms to prevail in certain life conditions. ENA genes found in the genome of the amoeba-flagellate *Naegleria gruberi* also point out the presence of ouabain-insensitive Na^+^-ATPase in this species. The structural characteristics of the ENA ATPase revealed that it maintains highly conserved sequences that are characteristic of P-type ATPases, such as the catalytic site, sequences involved in the nucleotide and Mg^2+^ binding, and others that are specific to ENA ATPases. *N. gruberi* shows the presence of the P type ATPase ouabain-insensitive and sensitive to furosemide identifying an ENA ATPase in the control of Na^+^^43^. The obtained results revealed that the Na^+^-ATPase ion pump of *N. fowleri* was ouabain-insensitive but sensitive to furosemide at concentrations of 0.71 ± 0.04 mM as mentioned in the “Results” section. Moreover, these data suggest that laurinterol (IC_50_ of 13,42 ± 2.57 µM) was able to inhibit this pump at concentrations 100 times lower than furosemide.

**Figure 12:**
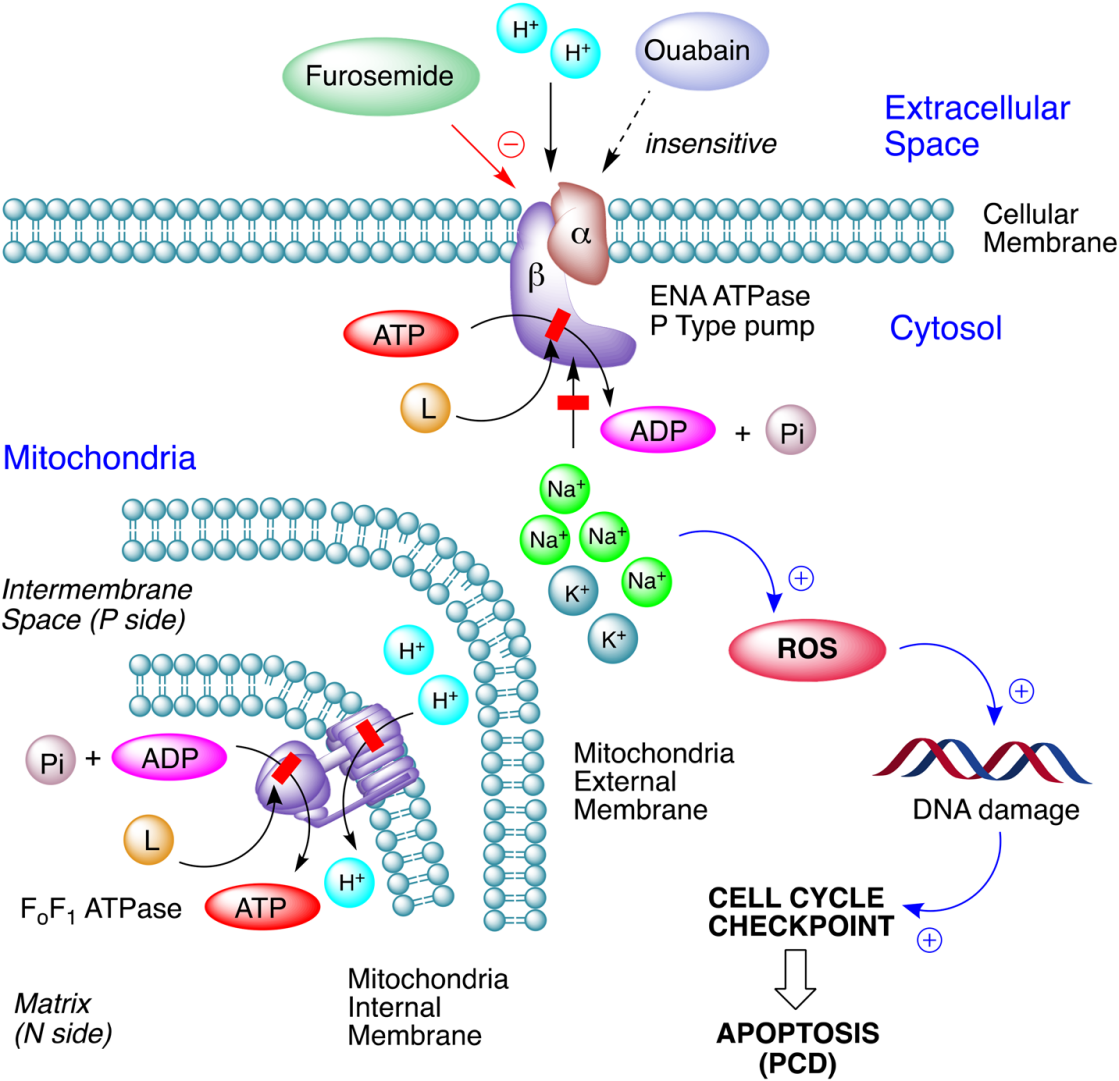
Proposed mechanism of action of laurinterol (**1**) as an ATP competitive inhibitor of P-type ATPases in *Naegleria fowleri.* **L** represents laurinterol (**1**).

Thus, due to the highly conserved sequences in the nucleotide binding pocket of this specific P-Type ATPase, a competitive inhibition by phenolic sequiterpenes such as laurinterol could explain all the observed effects in *N. fowleri* (Fig. 12).

**Figure 13:**
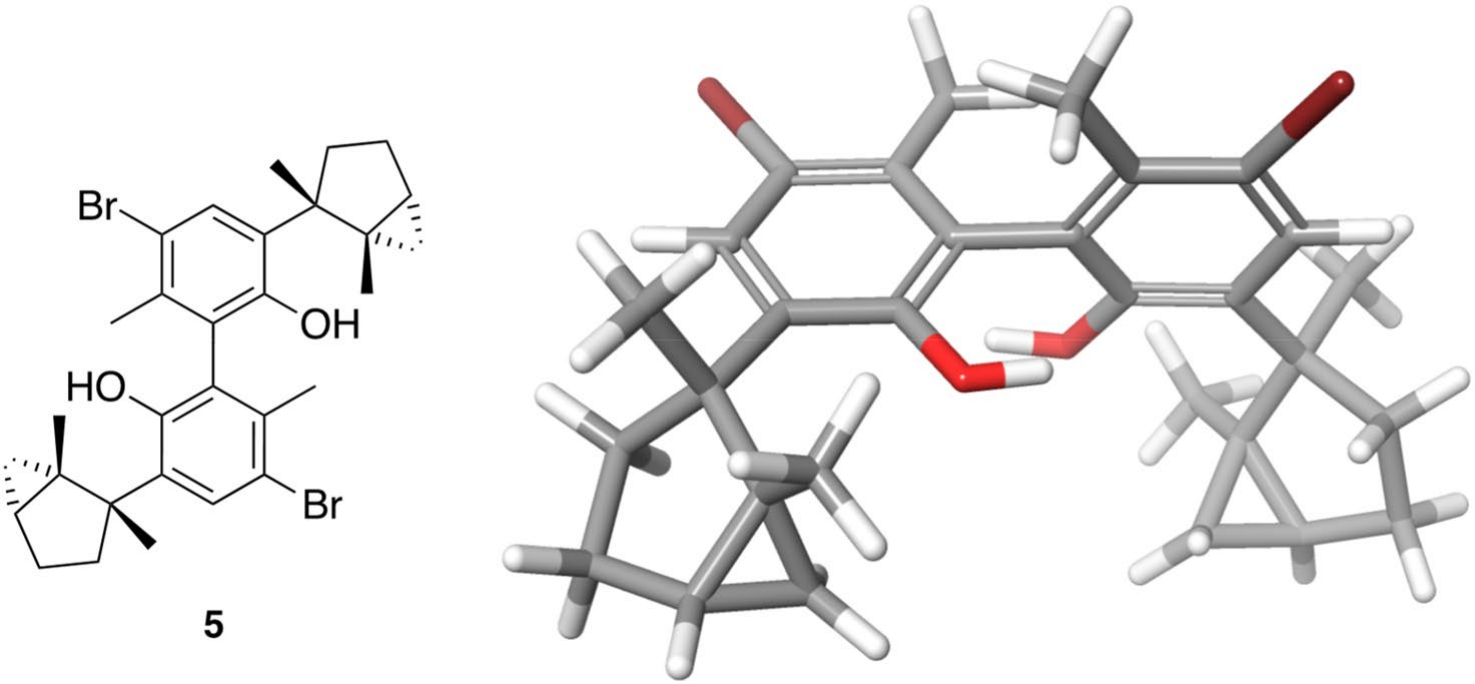
Structure and absolute configuration of laurinterol dimer (**5**) determined by X-ray crystallography.

Thus, an inhibition of sodium efflux ATPase would cause an increase in intracellular sodium concentration, which will trigger ROS, DNA damage and, hence, apoptosis^44–46^. At the level of cell organelles such as mitochondria, a plausible inhibition of FoF1 ATPase would cause a considerable decrease of ATP and strong changes in depolarization of the mitochondrial membrane^47^. All these effects caused by laurinterol in *N. fowleri* are consistent with a competitive, and not very specific, inhibition of ATPases. This assumption is based on the fact that laurinterol dimer (**5**) (Fig. 13) does not show activity (IC_50_ > 100 µM), possibly due to the increasement in volume of the molecule, which may limit the capacity to interact with the ATPases nucleotide binding pocket^38,39,43^. Nevertheless, further studies to evaluate this drug target as well as SARs approaches should be developed in the near future.

## Conclusion

In conclusion, laurinterol showed a high activity against *Naegleria fowleri* trophozoites. Moreover, laurinterol-treated amoebae showed signs of PCD as shown by DNA condensation, damages in cell membrane and mitochondria and ROS generation triggered by a possible inhibition of ENA ATPase ion pump. Therefore, laurinterol is presented as a novel, highly active, PCD inducer and low toxic anti-*Naegleria* compound which should be exploited for the development of novel therapeutic agents against PAM.

## Methods

### Collection and identification of *Laurencia johhstonii*

*Laurencia johnstonii* was collected by hand off the coast of Baja California Sur, Mexico (24° 21′ 10.8″ north (N), 110° 16′ 58.8″ west (W)). A voucher specimen (code 13-003) was deposited at the Herbarium of the Laboratory of Marine Algae of the Interdisciplinary Center of Marine Science (CICIMAR) and it was identified by Dr. R. Riosmena Rodríguez from the Autonomous University of Baja California Sur (UABCS).

### Extraction and isolation of laurinterol (1), debromolaurinterol (2) and isolaurinterol (3)

Washed and dried specimens of *L. johnstonii* were crushed and extracted with EtOH for three days at 25 °C. Dissolvent was replaced three times, the combined extracts were filtered through a Whatman no. 4 filter paper and concentrated under vacuum. The resulting extract was chromatographed in Sephadex LH-20 (500 × 70 mm, CH_3_OH, 100%) to obtain five fractions. The active fraction SF3 was separated in Flash silica gel (130 × 70 mm) using a stepwise gradient from *n*-hexane to ethyl acetate to obtain seven fractions. The active fraction SF3.2 (95% *n*-hexane) was chromatographed on a normal phase open column (300 × 50 mm) using a stepwise gradient from *n*-hexane to ethyl acetate to yield pure compounds **1**, **2** and **3**.

#### Laurinterol (1)

White crystal; [α]^25^_D_ + 17 (*c* 0.15, CH_2_Cl_2_); HRESIMS *m/z* 293.0531 [M−H]^−^ (calc. C_15_H_18_O^79^Br, 293.0541), 295.0518 [M−H]^−^ (calc. C_15_H_18_O^81^Br, 295.0521) ^1^H NMR (500 MHz, CDCl_3_) δ 0.55 (1H, dd, *J* = 7.9, 4.8 Hz, H-12), 0.58 (1H, t, *J* = 4.6 Hz, H-12), 1.15 (1H, dt, *J* = 8.1, 4.3 Hz, H-3), 1.28 (1H, m, H-5), 1.32 (3H, s, H-13), 1.41 (3H, s, H-14), 1.66 (1H, dd, *J* = 12.3, 8.0 Hz, H-4), 1.95 (1H, tdd, *J* = 12.3, 8.1, 4.4, H-4), 2.09 (1H, dd, *J* = 13.2, 8.1 Hz, H-5), 2.29 (3H, s, H-15), 5.26 (1H, br, s, 7-OH), 6,61 (1H, s, H-8), 7.61 (1H, s, H-11); ^13^C NMR (125 MHz, CDCl_3_) δ 16.2 (C-12), 18.6 (C-13), 22.2 (C-14), 23.5 (C-15), 24.4 (C-3), 25.3 (C-4), 29.6 (C-2), 35.9 (C-5), 114.9 (C-10), 118.8 (C-8), 132.3 (C-11), 134.0 (C-6), 135.9 (C-9), 153.3 (C-7).

#### Debromolaurinterol (2)

Colorless amorphous solid; [α]^25^_D_ -6.35 (*c* 0.19, CH_2_Cl_2_); HRESIMS *m/z* 215.1432 [M−H]^−^ (calc. C_15_H_19_O, 215.1436) ^1^H NMR (500 MHz, CDCl_3_) δ 0.52 (1H, dd, *J* = 7.8, 4.4 Hz, H-12), 0.59 (1H, t, *J* = 4.4 Hz, H-12), 0.91 (1H, m, H-3), 1.13 (1H, dt,* J* = 8.2, 4.1 Hz, H-5), 1.33 (3H, s, H-13), 1.43 (3H, s, H-14), 1.66 (1H, dd, *J* = 13.1, 8.4 Hz, H-4), 1.96 (1H, dddd, *J* = 12.3, 12.3, 8.0, 4.3 Hz, H-4), 2.13 (1H, dd, *J* = 13.1, 7.8 Hz, H-5), 2.28 (3H, s, H-15), 5.17 (1H, s, 7-OH), 6.56 (1H, d, *J* = 1.6 Hz, H-8), 6.70 (1H, dd, *J* = 7.9, 1.6 Hz, H-10), 7.39 (1H, d, *J* = 7.9 Hz, H-11); ^13^C NMR (125 MHz, CDCl_3_) δ 16.3 (C-12), 18.8 (C-13), 20.6 (C-14), 23.6 (C-15), 24.3 (C-3), 25.3 (C-4), 27.7 (C-2), 36.0 (C-5), 48.0 (C-1), 117.3 (C-10), 120.8 (C-8), 128.8 (C-11), 131.4 (C-6), 136.7 (C-9), 154.0 (C-7).

#### Isolaurinterol (3)

Colorless amorphous solid; [α]^25^_D_ -46 (*c* 0.14, CH_2_Cl_2_); HRESIMS *m/z* 293.0536 [M−H]^−^ (calc. C_15_H_18_O^79^Br, 293.0541), 295.0528 [M—H]^-^ (calc. C_15_H_18_O^81^Br, 295.0521) ^1^H NMR (500 MHz, CDCl_3_) δ 1.21 (3H, d, *J* = 7.0 Hz, H-12), 1.42 (1H, ddd, *J* = 12.8, 8.2, 6.6 Hz, H-5), 1.46 (3H, s, H-14), 1.59 (1H, dt, *J* = 12.9, 7.1, 7.1 Hz, H-4), 2.05 (1H, ddt *J* = 12.8, 8.5, 7.0, 7.0 Hz, H-3), 2.20 (1H, ddd, *J* = 13.0, 8.1 6.7 Hz, H-4), 2.31 (3H, s, H-15), 2.85 (1H, ddt, *J* = 9.1, 6.9, 6.9, 2.3, 2.3 Hz, H-5), 4.94 (1H, d, *J* = 2.2 Hz, H-13), 5.11 (1H, d, *J* = 2.2 Hz, H-13), 5.56 (1H, br, s, 7-OH), 6.73 (1H, s, H-8), 7.45 (1H, s, H-11); ^13^C NMR (125 MHz, CDCl_3_) δ 21.2 (C-14), 22.2 (C-15), 27.8 (C-12), 31.2 (C-4), 37.6 (C-3), 39.1 (C-5), 49.8 (C-1), 106.9 (C-13), 115.5 (C-10), 120.4 (C-8), 131.2 (C-11), 132.7 (C-6), 137.2 (C-9), 153.0 (C-7), 165.4 (C-2).

### Synthesis of laurinterol dimer (5)

Laurinterol dimer (**5**) was obtained by oxidation of laurinterol according to Ichiba and Higa (1986) with some modifications. Briefly, **1** (50 mg) was dissolved in 80% acetic acid (1 mL) and 200 μL of 25% chromium trioxide solution in acetic acid was added. The reaction was left under magnetic stirring for 2 h at 4 °C and extracted with dichloromethane after addition of water. The reaction mixture was separated on a normal phase open column (Silicagel, 0.2−0.5 mm, 30 Ø × 110 mm) eluted with *n*-Hex/EtOAc (99:1) to obtain 7 fractions. Fraction 2 was purified by HPLC on a normal phase column (Luna 5 µm Silica 100 Å, 10 Ø × 250 mm) eluted with *n*-Hex/EtOAc (99:1) to yield **5**, (2 mg).

Laurinterol dimer (**5**). White crystal; [α]^25^_D_—32.5 (*c* 0.04, CH_2_Cl_2_); HRESIMS *m/z* 585.1000 [M−H]^−^ (calc. C_30_H_35_O_2_^79^Br_2_, 293.0541), 587.0981 [M−H]^−^ (calc. C_30_H_35_O_2_^79^Br ^81^Br, 587.0983), 589.0951 [M^−^H]^−^ (calc. C_30_H_35_O_2_^81^Br_2_, 589.0963); ^1^H NMR (500 MHz, CDCl_3_) δ 0.54 (1H, d, *J* = 5.3 Hz, H-13), 0.57 (1H, d, *J* = 5.1 Hz, H-13), 1.11 (1H, dt, *J* = 8.1, 4.2 Hz, H-3), 1.26 (2H, m, H-5), 1.33 (3H, s, H-12), 1.38 (3H, s, H-14), 1.63 (1H, dd, *J* = 12.4, 7.9 Hz, H-4), 1.92 (1H, m, H-4), 2.02 (3H, s, H-15), 2.18 (1H, m, H-5), 4.77 (1H, br, s, 7-OH), 7.83 (1H, s, H-11); ^13^C NMR (500 MHz, CDCl_3_) δ 16.3 (C-13), 18.9 (C-12), 19.9 (C-15), 22.5 (C-14), 24.3 (C-3), 25.4 (C-4), 29.7 (C-2), 35.5 (C-5), 48.7 (C-1), 115.9 (C-10), 122.0 (C-8), 133.3 (C-11), 135.1 (C-9), 135.6 (C-6), 152.1 (C-7). NMR data are in accordance with those previously reported for the natural compound^49^. X-Ray crystallographic data of **5** is reported for the first time (Supplementary material S1). CCDC 2036372 contains the supplementary crystallographic data for this paper. These data can be obtained free of charge from The Cambridge Crystallographic Data Centre via www.ccdc.cam.ac.uk/structures^50^.

### Commercial ATPase ion pump inhibitors

Ouabain octahydrate (CAS no. 11018-89-6) and furosemide (CAS no. 54-31-9) were acquired from Sigma-Aldrich.

### In vitro amoebicidal and cytotoxicity assays

#### *Naegleria fowleri* strain

A type strain of *Naegleria fowleri* with reference number ATCC 30808 from the American Type Culture Collection (LG Promochem, Barcelona, Spain) was used in this study. The strain was axenically cultured at 37 ºC in 2% (w/v) Bactocasitone medium (Thermo Fisher Scientific, Madrid, Spain) supplemented with 10% (v/v) foetal bovine serum (FBS), containing 0,5 mg/ml of Streptomycin sulfate (Sigma-Aldrich, Madrid, Spain) and 0.3 µg/ml of Penicillin G Sodium Salt (Sigma-Aldrich, Madrid, Spain). The strains were cultured in a biological security facility of level 3 at our institution following Spanish biosafety guidelines for this pathogen.

### In vitro activity assays against the trophozoite stage of *Naegleria fowleri*

The activities of the tested compounds against *Naegleria fowleri* trophozoites were determined using a colorimetric assay as previously described^9^. Briefly, *Naegleria* trophozites were seeded in duplicate on a 96-well microtiter plate with 50 μL from a stock solution of 2 × 10^5^ cells/ml. After that, a serial dilution of the evaluated compounds (in the same culture medium as the amoeba) was added to the plate. A negative control in the plates, consisted in amoebae in medium alone. Finally, the AlamarBlue reagent (Life Technologies, Madrid, Spain) was placed in each well (10% of medium volume) and plates were incubated with slight agitation for 48 h at 37 °C. After that, plates were analysed with an EnSpire Multimode Plate Reader (Perkin Elmer, Madrid, Spain) using a wavelength of excitation of 570 nm and a wavelength of emission of 585 nm. To calculate the percentages of growth inhibition and the 50% and 90% inhibitory concentrations (IC_50_ and IC_90_) a no linear regression analysis was performed with a 95% confidence limit using the SigmaPlot 12.0 software (Systat Software Inc.,London, UK). Experiments were performed in triplicated and the mean values were also calculated. A paired two-tailed *t*-test was used for the analysis of the data and the values of P < 0.05 were considered statistically significant. In all assays, 1% DMSO was used to dissolve the highest dose of the compounds. When 1% DMSO was added to medium alone (without any investigated compound) no effect was observed on the amoebae.

### Cytotoxicity assays

Cytotoxicity of laurinterol (the most active tested compound) was evaluated after 24 h incubation of a murine macrophage J774.A1 cell line (ATCC # TIB-67) with different concentration of the tested compound at 37 ˚C in a 5% CO_2_ humidified incubator as previously described^51^.

### Double-stain assay for programmed cell death determination

A double-stain apoptosis detection kit (Hoechst 33342/PI) (Life Technologies, Madrid, Spain) and an EVOS FL Cell Imaging System AMF4300, (Life Technologies, Madrid, Spain) were used in this assay. The experiment was carried out by following the manufacturer’s recommendations, and 5 × 10^5^ cells/ml well were incubated in a 96-well plate for 24 h with the previously calculated IC_90_. As it has been previously reported^52^, the double-staining pattern allows the identification of three groups in a cellular population: live cells will show only a low level of fluorescence, cells undergoing PCD will show a higher level of blue fluorescence (as chromatin condenses), and dead cells will show low-blue and high-red fluorescence (as the Propidium Iodide stain enters the nucleus).

### CellROX deep red staining

The generation of intracellular ROS was evaluated by using the CellROX Deep Red fluorescent probe (Invitrogen, Termo Fisher Scientific, Madrid, Spain). *Naegleria fowleri* trophozoites were treated with the IC_90_ of laurinterol for 24 h and exposed to CellROX Deep Red (5 μM, 30 min) at 37 °C in the dark. Cells were observed in an EVOS FL Cell Imaging System AMF4300 (Life Technologies, Madrid, Spain).

### Analysis of mitochondrial function disruption

#### Mitochondrial membrane potential

The collapse of an electrochemical gradient across the mitochondrial membrane during PCD induction in treated amoebae was detected with the JC-1 mitochondrial membrane potential detection kit (Cayman Chemicals, Vitro SA, Madrid, Spain). Amoebae were incubated with the IC_90_ of laurinterol for 24 h and the experiment was carried out by following the manufacturer’s recommendations. Images were captured using an EVOS FL Cell Imaging System AMF4300, Life Technologies, Madrid, Spain. The obtained staining pattern allowed the identification of two groups in a cellular population: live cells will show only red fluorescence; cells with low mitochondrial potential, (undergoing PCD) will show a higher level of green and red fluorescence.

#### ATP levels

ATP levels were measured using a CellTiter-Glo Luminescent Cell Viability Assay (PROMEGA BIOTECH IBÉRICA S.L, Madrid, Spain). The effect of the drug on the ATP production was evaluated by incubating 5 × 10^5^ cells/ml with the previously calculated IC_90_ of laurinterol.

#### Plasma membrane permeability

The SYTOX Green assay was performed to detect alterations of the membrane permeability in treated cells. Briefly, 5 × 10^5^ trophozoites were incubated with the previously calculated IC_90_ of laurinterol. After 24 h of incubation, the SYTOX Green was added at a final concentration of 1 μM (Molecular Probes). Cells were observed after 15 min in an EVOS FL Cell Imaging System AMF4300 (Life Technologies, Madrid, Spain).

## Supplementary information

Supplementary file 1
