# Supplementary material for: Laurinterol from Laurencia johnstonii eliminates Naegleria fowleri triggering PCD by inhibition of ATPases
Source: Sci Rep. 2020 Oct 20;10:17731. doi: 10.1038/s41598-020-74729-y (PMC7576160; doi:10.1038/s41598-020-74729-y)
Supplement: Supplementary file 1 [file 41598_2020_74729_MOESM1_ESM.pdf]

SUPPLEMENTARY INFORMATION

Laurinterol from *Laurencia johnstonii* eliminates *Naegleria fowleri* triggering PCD by inhibition of ATPases

Iñigo Arberas-Jiménez[1,2†], Sara García-Davis[3,†], Aitor Rizo-Liendo[1,2], Ines Sifaoui[1,4], María Reyes-Batlle[1,2], Olfa Chiboub[1,4], Rubén L. Rodríguez-Expósito[1,2], Ana R. Díaz-Marrero[3], José E. Piñero[1,2]*, José J. Fernández[3,5]*,and Jacob Lorenzo-Morales [1,2]*

[1] Instituto Universitario de Enfermedades Tropicales y Salud Pública de Canarias, Universidad de La Laguna. Av. Astrofísico Francisco Sánchez S/N, 38203, Tenerife, España
[2] Departamento de Obstetricia y Ginecología, Pediatría, Medicina Preventiva y Salud Pública, Toxicología, Medicina Legal y Forense y Parasitología, Universidad de La Laguna, Avda. Astrofísico F. Sánchez s/n, 38206 La Laguna, Tenerife, Spain
[3] Instituto Universitario de Bio-Orgánica Antonio González (IUBO AG), Universidad de La Laguna (ULL), Avda. Astrofísico F. Sánchez, 2, 38206 La Laguna, Tenerife, Spain
[4] Laboratoire Matériaux-Molécules et Applications, La Marsa, University of Carthage, Tunisia, Tunisia
[5] Departamento de Química Orgánica, Universidad de La Laguna (ULL), Avda. Astrofísico F. Sánchez, 2, 38206 La Laguna, Tenerife, Spain
* Correspondence and requests for materials should be addressed to J.L.M. (jmlorenz@ull.edu.es); J.E.P.B. (jpinero@ull.edu.es) or J.F.F. (jjfercas@ull.edu.es)
† Both authors contributed equally to this work.

TABLE OF CONTENTS

CONTENTS	PAGE
**Figure S1. Top:** Isolation procedure for compounds 1-3 **Bottom: A.** Crude extract of *Laurencia johnstonii;* **B.** Sephadex LH20 chromatography column; **C.** Chromatography fractions of silica gel columns	S3
**Figure S2.** ^{1}H-NMR spectrum of laurinterol dimer (**5**) $CDCl_3$ at 299 K, 500 MHz.	S4
**Figure S3.** ^{13}C spectrum of laurinterol dimer (**5**) $CDCl_3$ at 299 K, 500 MHz.	S4
**Table S1.** Crystal structure and data for laurinterol dimer (**5**)	S6
**Table S2.** Atomic coordinates for laurinterol dimer (**5**)	S7
**Table S3.** Bond lengths for laurinterol dimer (**5**)	S9
**Table S4.** Bond angles for laurinterol dimer (**5**)	S10

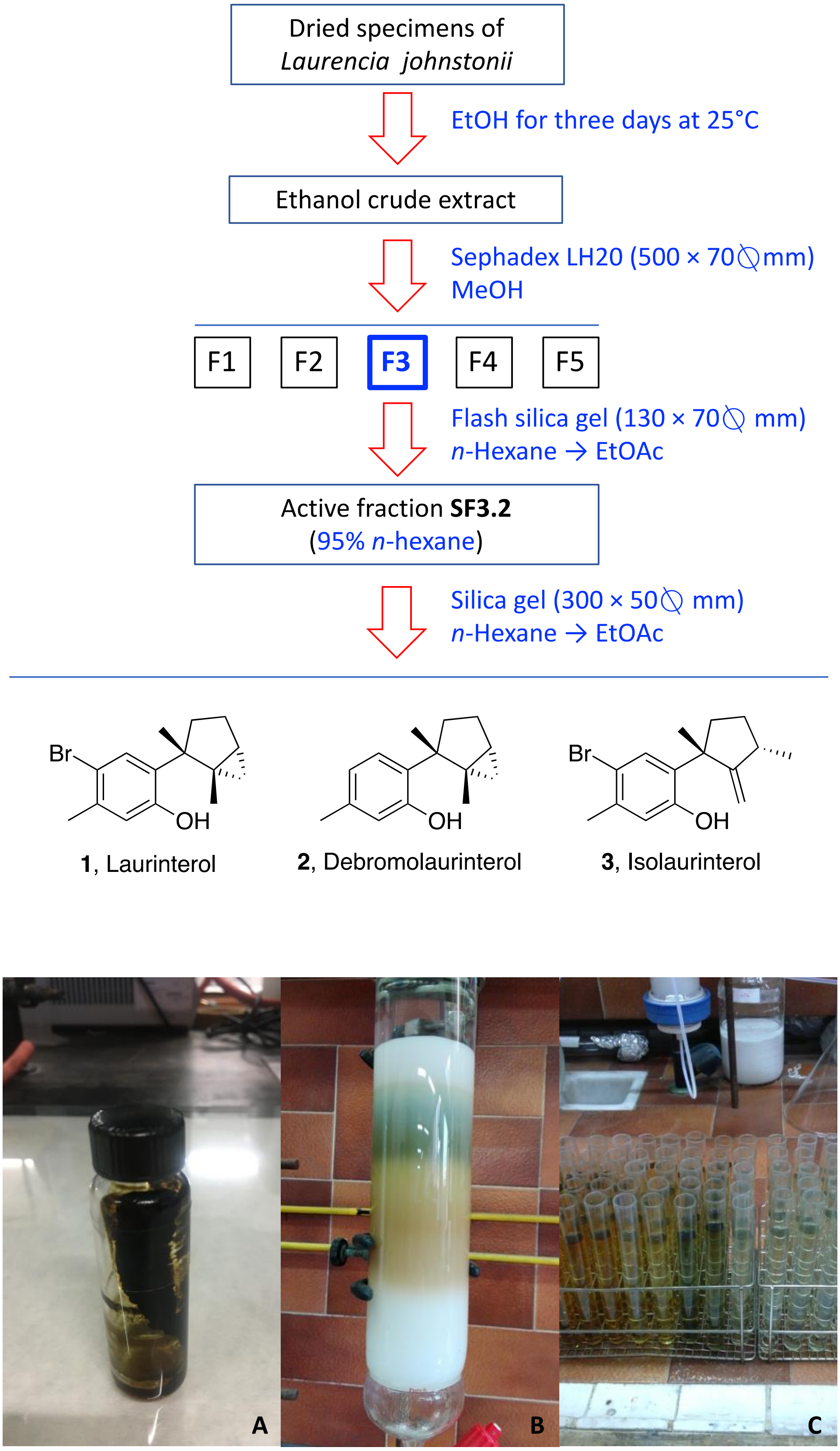

Figure S1. Top: Isolation procedure for compounds **1**-**3**. **Bottom: A.** Crude extract of *Laurencia johnstonii;* **B.** Sephadex LH20 chromatography column; **C.** Chromatography fractions of silica gel columns.

Figure S2. ^{1}H-NMR of laurinterol dimer (**5**) $CDCl_3$ at 299 K, 500 MHz.

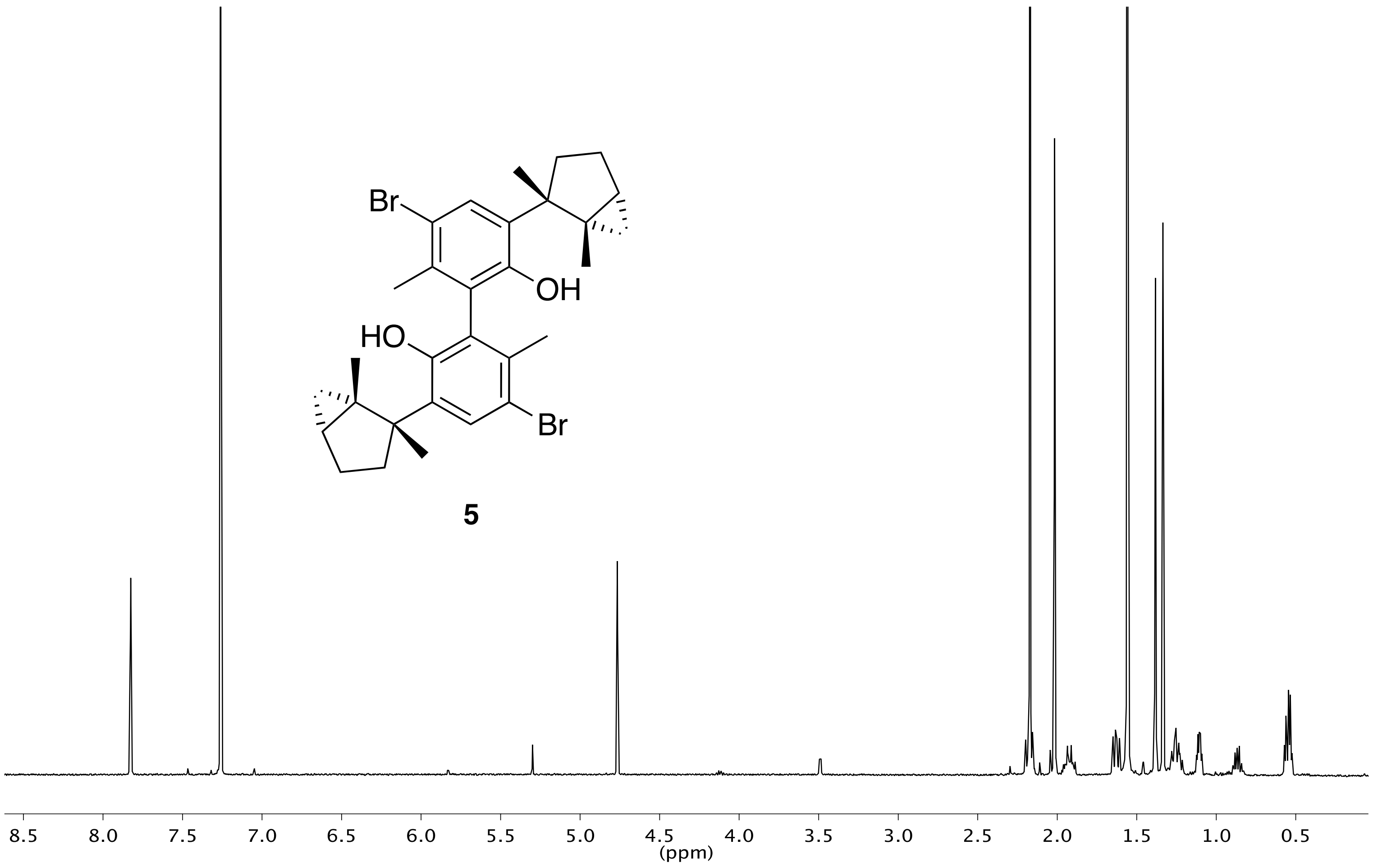

Figure S3. ^{13}C spectrum of laurinterol dimer (**5**) $CDCl_3$ at 299 K, 500 MHz.

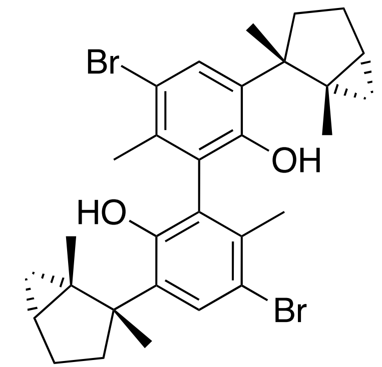

5

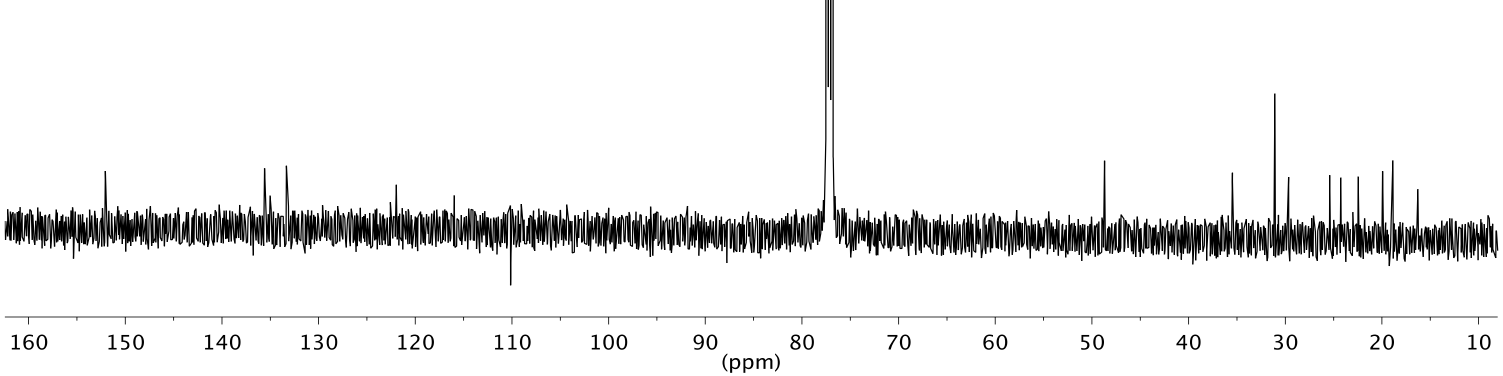

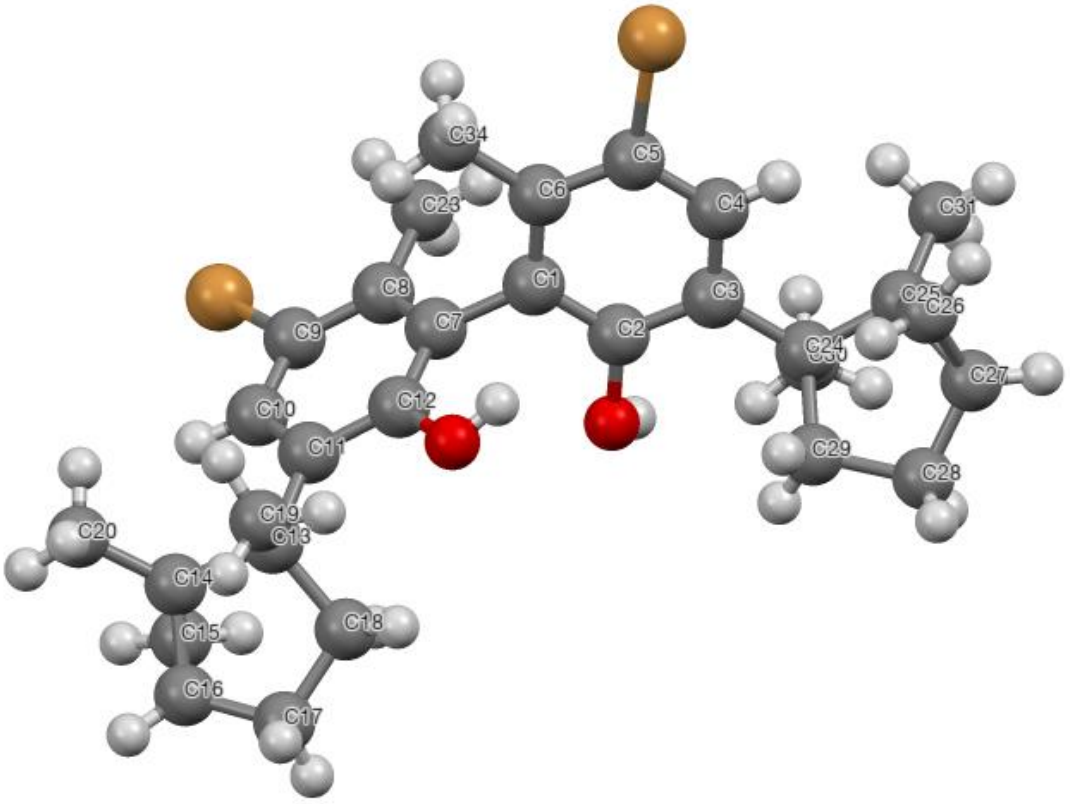

Table S1. Crystal structure and data for laurinterol dimer (**5**).

**Identification code**	SG-004
**Empirical formula**	$C_{30} H_{36} Br_2 O_2$
**Crystal system**	Monoclinic
**Space group**	$P\ 2_1$
**Cell_length_a**	6.3618(2)
**Cell_length_b**	15.7169(4)
**Cell_length_c**	13.9734(3)
**Cell_angle_alpha**	90.0
**Cell_angle_beta**	95.061(3)
**Cell_angle_gamma**	90.0
**Cell_volume**	1391.72(7)
**Cell_formula_units_Z**	2

Table S2. Atomic coordinates for laurinterol dimer (**5**).

Atom	SybylType	Xfrac + ESD	Yfrac + ESD	Zfrac + ESD	Symm. op.
BR21	Br	1,013590	0,387568	1,018060	x,y,z
BR32	Br	0,493527	0,222781	0,463961	x,y,z
O22	O.3	0,468453	0,552399	0,697891	x,y,z
H22	H	0,467835	0,523952	0,648674	x,y,z
O33	O.3	0,942674	0,536106	0,619172	x,y,z
H33	H	1,070530	0,529802	0,618703	x,y,z
C3	C.2	0,859873	0,440190	0,490484	x,y,z
C4	C.2	0,752085	0,367646	0,458426	x,y,z
H4	H	0,758721	0,350071	0,395212	x,y,z
C12	C.2	0,589984	0,512848	0,770309	x,y,z
C11	C.2	0,581384	0,543925	0,864317	x,y,z
C24	C.3	0,991448	0,491543	0,424377	x,y,z
C10	C.2	0,707708	0,502566	0,935814	x,y,z
H10	H	0,705440	0,520054	0,999296	x,y,z
C9	C.3	0,837177	0,435649	0,914221	x,y,z
C6	C.2	0,616289	0,342820	0,613561	x,y,z
C13	C.3	0,451240	0,622222	0,885898	x,y,z
C2	C.2	0,841885	0,463740	0,586251	x,y,z
C1	C.2	0,723376	0,416115	0,646616	x,y,z
C8	C.2	0,849813	0,404940	0,822393	x,y,z
C5	C.3	0,634842	0,320787	0,518339	x,y,z
C7	C.2	0,721141	0,444577	0,749466	x,y,z
C25	C.3	0,976901	0,457831	0,320360	x,y,z
C18	C.3	0,535195	0,701399	0,834788	x,y,z
H18A	H	0,686520	0,697344	0,831461	x,y,z
H18B	H	0,467499	0,706493	0,770074	x,y,z
C19	C.3	0,216866	0,609433	0,854497	x,y,z
H19A	H	0,195919	0,609337	0,785614	x,y,z
H19B	H	0,136778	0,654858	0,879394	x,y,z
H19C	H	0,170837	0,556079	0,878780	x,y,z
C34	C.3	0,485740	0,291192	0,676947	x,y,z
H34A	H	0,552931	0,237318	0,690580	x,y,z
H34B	H	0,347972	0,282066	0,644749	x,y,z
H34C	H	0,472997	0,321242	0,735968	x,y,z
C29	C.3	0,908144	0,583912	0,412038	x,y,z
H29A	H	0,756388	0,585476	0,414846	x,y,z
H29B	H	0,973544	0,620291	0,462255	x,y,z
C26	C.3	0,774883	0,476689	0,260247	x,y,z
H26A	H	0,736077	0,439695	0,205984	x,y,z
H26B	H	0,657111	0,498137	0,292722	x,y,z

C15	C.3	0,681153	0,688848	1,028480	x,y,z
H15A	H	0,796561	0,686173	0,987608	x,y,z
H15B	H	0,723056	0,685846	1,096840	x,y,z
C14	C.3	0,474794	0,648219	0,992228	x,y,z
C20	C.3	0,354452	0,601438	1,064840	x,y,z
H20A	H	0,205707	0,609546	1,049570	x,y,z
H20B	H	0,394507	0,623277	1,127990	x,y,z
H20C	H	0,386855	0,541837	1,063000	x,y,z
C31	C.3	1,103190	0,380905	0,296844	x,y,z
H31A	H	1,250610	0,394983	0,302558	x,y,z
H31B	H	1,061450	0,362903	0,232306	x,y,z
H31C	H	1,078140	0,335727	0,340656	x,y,z
C16	C.3	0,493435	0,743859	0,995842	x,y,z
H16	H	0,421683	0,775051	1,044230	x,y,z
C17	C.3	0,481067	0,777388	0,895700	x,y,z
H17A	H	0,581383	0,823235	0,890245	x,y,z
H17B	H	0,340348	0,798262	0,876086	x,y,z
C23	C.3	0,994465	0,332769	0,801041	x,y,z
H23A	H	1,138550	0,350724	0,813355	x,y,z
H23B	H	0,968168	0,316531	0,734838	x,y,z
H23C	H	0,968830	0,285075	0,841329	x,y,z
C30	C.3	1,224100	0,492544	0,464159	x,y,z
H30A	H	1,238430	0,523581	0,523548	x,y,z
H30B	H	1,307040	0,519459	0,418615	x,y,z
H30C	H	1,272390	0,435217	0,475201	x,y,z
C27	C.3	0,960639	0,533799	0,254297	x,y,z
H27	H	1,035740	0,532532	0,196023	x,y,z
C28	C.3	0,967005	0,613407	0,312869	x,y,z
H28A	H	0,866147	0,654769	0,285104	x,y,z
H28B	H	1,106850	0,638487	0,317487	x,y,z

Table S3. Bond lengths for laurinterol dimer (**5**).

Atom1	Atom2	Cyclicity	Length
BR21	C9	acyclic	1,9102
BR32	C5	acyclic	1,9073
O22	H22	acyclic	0,8200
O22	C12	acyclic	1,3675
O33	H33	acyclic	0,8200
O33	C2	acyclic	1,3654
C3	C4	cyclic	1,3844
C3	C24	acyclic	1,5303
C3	C2	cyclic	1,4028
C4	H4	acyclic	0,9300
C4	C5	cyclic	1,3820
C12	C11	cyclic	1,4071
C12	C7	cyclic	1,4054
C11	C10	cyclic	1,3874
C11	C13	acyclic	1,5280
C24	C25	cyclic	1,5421
C24	C29	cyclic	1,5499
C24	C30	acyclic	1,5345
C10	H10	acyclic	0,9300
C10	C9	cyclic	1,3854
C9	C8	cyclic	1,3799
C6	C1	cyclic	1,3961
C6	C5	cyclic	1,3900
C6	C34	acyclic	1,5042
C13	C18	cyclic	1,5528
C13	C19	acyclic	1,5299
C13	C14	cyclic	1,5355
C2	C1	cyclic	1,3973
C1	C7	acyclic	1,5064
C8	C7	cyclic	1,3962
C8	C23	acyclic	1,5070
C25	C26	cyclic	1,5016
C25	C31	acyclic	1,5039
C25	C27	cyclic	1,5072
C18	H18A	acyclic	0,9700
C18	H18B	acyclic	0,9700
C18	C17	cyclic	1,5235
C19	H19A	acyclic	0,9600

Atom1	Atom2	Cyclicity	Length
C19	H19B	acyclic	0,9600
C19	H19C	acyclic	0,9600
C34	H34A	acyclic	0,9600
C34	H34B	acyclic	0,9600
C34	H34C	acyclic	0,9600
C29	H29A	acyclic	0,9700
C29	H29B	acyclic	0,9700
C29	C28	cyclic	1,5385
C26	H26A	acyclic	0,9700
C26	H26B	acyclic	0,9700
C26	C27	cyclic	1,4921
C15	H15A	acyclic	0,9700
C15	H15B	acyclic	0,9700
C15	C14	cyclic	1,5068
C15	C16	cyclic	1,5118
C14	C20	acyclic	1,5144
C14	C16	cyclic	1,5083
C20	H20A	acyclic	0,9600
C20	H20B	acyclic	0,9600
C20	H20C	acyclic	0,9600
C31	H31A	acyclic	0,9600
C31	H31B	acyclic	0,9600
C31	H31C	acyclic	0,9600
C16	H16	acyclic	0,9800
C16	C17	cyclic	1,4908
C17	H17A	acyclic	0,9700
C17	H17B	acyclic	0,9700
C23	H23A	acyclic	0,9600
C23	H23B	acyclic	0,9600
C23	H23C	acyclic	0,9600
C30	H30A	acyclic	0,9600
C30	H30B	acyclic	0,9600
C30	H30C	acyclic	0,9600
C27	H27	acyclic	0,9800
C27	C28	cyclic	1,4937
C28	H28A	acyclic	0,9700
C28	H28B	acyclic	0,9700

Table S4. Bond angles for laurinterol dimer (**5**).

Atom1	Atom2	Cyclicity	Length
BR21	C9	acyclic	1,9102
BR32	C5	acyclic	1,9073
O22	H22	acyclic	0,8200
O22	C12	acyclic	1,3675
O33	H33	acyclic	0,8200
O33	C2	acyclic	1,3654
C3	C4	cyclic	1,3844
C3	C24	acyclic	1,5303
C3	C2	cyclic	1,4028
C4	H4	acyclic	0,9300
C4	C5	cyclic	1,3820
C12	C11	cyclic	1,4071
C12	C7	cyclic	1,4054
C11	C10	cyclic	1,3874
C11	C13	acyclic	1,5280
C24	C25	cyclic	1,5421
C24	C29	cyclic	1,5499
C24	C30	acyclic	1,5345
C10	H10	acyclic	0,9300
C10	C9	cyclic	1,3854
C9	C8	cyclic	1,3799
C6	C1	cyclic	1,3961
C6	C5	cyclic	1,3900
C6	C34	acyclic	1,5042
C13	C18	cyclic	1,5528
C13	C19	acyclic	1,5299
C13	C14	cyclic	1,5355
C2	C1	cyclic	1,3973
C1	C7	acyclic	1,5064
C8	C7	cyclic	1,3962
C8	C23	acyclic	1,5070
C25	C26	cyclic	1,5016
C25	C31	acyclic	1,5039
C25	C27	cyclic	1,5072
C18	H18A	acyclic	0,9700
C18	H18B	acyclic	0,9700
C18	C17	cyclic	1,5235
C19	H19A	acyclic	0,9600

Atom1	Atom2	Cyclicity	Length
C19	H19B	acyclic	0,9600
C19	H19C	acyclic	0,9600
C34	H34A	acyclic	0,9600
C34	H34B	acyclic	0,9600
C34	H34C	acyclic	0,9600
C29	H29A	acyclic	0,9700
C29	H29B	acyclic	0,9700
C29	C28	cyclic	1,5385
C26	H26A	acyclic	0,9700
C26	H26B	acyclic	0,9700
C26	C27	cyclic	1,4921
C15	H15A	acyclic	0,9700
C15	H15B	acyclic	0,9700
C15	C14	cyclic	1,5068
C15	C16	cyclic	1,5118
C14	C20	acyclic	1,5144
C14	C16	cyclic	1,5083
C20	H20A	acyclic	0,9600
C20	H20B	acyclic	0,9600
C20	H20C	acyclic	0,9600
C31	H31A	acyclic	0,9600
C31	H31B	acyclic	0,9600
C31	H31C	acyclic	0,9600
C16	H16	acyclic	0,9800
C16	C17	cyclic	1,4908
C17	H17A	acyclic	0,9700
C17	H17B	acyclic	0,9700
C23	H23A	acyclic	0,9600
C23	H23B	acyclic	0,9600
C23	H23C	acyclic	0,9600
C30	H30A	acyclic	0,9600
C30	H30B	acyclic	0,9600
C30	H30C	acyclic	0,9600
C27	H27	acyclic	0,9800
C27	C28	cyclic	1,4937
C28	H28A	acyclic	0,9700
C28	H28B	acyclic	0,9700
